# Prediction of Congou Black Tea Fermentation Quality Indices from Color Features Using Non-Linear Regression Methods

**DOI:** 10.1038/s41598-018-28767-2

**Published:** 2018-07-12

**Authors:** Chunwang Dong, Gaozhen Liang, Bin Hu, Haibo Yuan, Yongwen Jiang, Hongkai Zhu, Jiangtao Qi

**Affiliations:** 1 Tea Research Institute Chinese Academy of Agricultural Sciences, Key Laboratory of Tea Biology and Resources Utilization, Ministry of Agriculture, Hangzhou, 310008 China; 2 College of Mechanical and Electrical Engineering, Shihezi University, Shihezi, 832003 China; 3 Department of Food Science, University of Copenhagen, Frederiksberg, 999017 Denmark

## Abstract

Fermentation is the key process to produce the special color of congou black tea. The machine vision technology is applied to detect the color space changes of black tea’s color in RGB, Lab and HSV, and to find out its relevance to black tea’s fermentation quality. And then the color feature parameter is used as input to establish physicochemical indexes (TFs, TRs, and TBs) and sensory features’ linear and non-linear quantitative evaluation model. Results reveal that color features are significantly correlated to quality indices. Compared with the other two color models (RGB and HSV), CIE Lab model can better reflect the dynamic variation features of quality indices and foliage color information of black tea. The predictability of non-linear models (RF and SVM) is superior to PLS linear model, while RF model presents a slight advantage over the classic SVM model since RF model can better represent the quantitative analytical relationship between image information and quality indices. This research has proved that computer image color features and non-linear method can be used to quantitatively evaluate the changes of quality indices (e.g. sensory quality) and the pigment during black tea’s fermentation. Besides, the test is simple, fast, and nondestructive.

## Introduction

Black tea is one of the most popular functional beverages in the world; based on the shape, it can be classified into two categories: broken black tea and bar shaped tea (Congou black tea)^1^. Black tea is a kind of fully fermented tea. Color and odor are important sensory changing during the process of black tea fermentation. During the fermentation, catalyzed by polyphenol oxidase (PPO) and peroxidase, tea polyphenol (especially catechin component) will gradually produce colored oxidation products pigment substances, among which water-soluble pigment substances have strong influence on black tea’s sensory quality^2,3^. These water-soluble pigment substances mainly include theaflavins (TFs), thearubigins (TRs), and theabrownin (TBs)^3^.

The color and luster of tea soup will be formed as the above pigment substances dissolve in water; the dissolution of pigment substances will also influence the taste and flavor of tea soup, such as sweet taste, salty taste, intensity, concentration, and refreshing degree^4,5^. TFs are chemical compounds with benzotropolone structure and in golden yellow. As TFs dissolve in water, the tea soup will present a visual feature of “shiny”; meanwhile, a special “golden ring” of black tea will form at the edge of the tea soup that contacts with the teacup^6^. The content of TFs is in positive correlation with the brightness of tea soup. The taste of TFs is pungent with strong astringency, which is an important component of tea soup’s taste intensity and refreshing degree. TRs are complicated bronzing phenols with very large relative molecular mass difference. TRs will turn crimson when dissolve in water, which is also an important component of tea soup’s concentration and intensity. TRs taste sweet and mellow, which are less pungent and intense than TFs. TRs have positive influence on tea soup’s ruby red color, but too much TRs will darken the color of tea soup and affect the sensory quality. TBs is a kind of dark brown high polymer compound that is water-soluble and non-dialytic; it is mainly produced from the oxidation of TRs and TFs. TBs is the main cause of somber tea soup; it has negative correlation with black tea’s soup color, infused leaf, taste, and other sensory quality^7^.

Moreover, the black tea leaves will have distinct color changes during fermentation, changing from turquoise to yellowish green, and then to yellowish red, yellowish brown, and finally to dark brown. This color changing process is defined as “red stain” in tea manufacture^6,8,9^. From the perspective of tea leaf biochemistry, red stain is a result of pigment’s dynamic transition from polyphenols to TFs, TRs, and TBs^3,10^. This kind of color change can be observed and distinguished by human visual system, but it is very hard to determine the specific scale^11^. The sensory description of human to color is qualitative. However, in large-scale production of black tea, the fermentation degree and sensory quality can be predicted through rich manufacture experience and the observation of the above color changes. For this reason, the fermentation could be insufficient or excessive, and the color of tea leaf could be mixed and uneven; besides, the flavor of made tea could be unpredictable.

Color is an important attribute and perception feature of computer image. The color information of black tea leaves can be quantified and accurately described by capturing sample images with machine vision acquisition system and extracting the color features with digitization^12,13^. Visible light images, hyper-spectrum, and near-infrared technology have already been used by researchers to identify tea’s category, quality, shape, and place of origin^9,14^. However, there are few researches about the quality detection technology for black tea’s fermentation process^15,16^. Surajit Borah *et al*.^17,18^ established a detection system, which can be used to detect the fermentation degree of broken black tea. In this system, images of tea leaves are collected intermittently during fermentation; HIS and RGB channel histograms are extracted; the image with the highest sensory scores will be defined as the standard image of its group; then, the distance (DPV value) between the measured image and the standard image can be obtained based on Manhattan distance algorithm; for any sample whose DPV values of three RGB channels are lower than 0.3, it can be regarded as properly fermented. Mohit Sharma *et al*.^6^ conducted research into the RGB color change differences between materials with varied rolling fineness degrees during fermentation. Their findings reveal that larger grain size will result in lower oxidation rate, and longer color change and transference cycle. Yudong Zhang based on using a novel fractional fourier entropy and jaya algorithm designed a tea-category identification (TCI) system, which can automatically determine tea category from images captured by a 3 charge-coupled device (CCD) digital camera^19^. Xueyan Wu based on optimal wavelet entropy and weighted k-Nearest Neighbors algorithm developed a tea-category identification system based on machine learning and computer vision with the aim of classifying different tea types automatically and accurately^20^. Gurpreet Singh^21^ proposed the appearance integrated quality indices TQI (appropriate weights to the grain diameter, perimeter, area and average color) using the color information (RGB and grey level) of the image; this evaluation method can well distinguish the quality difference of fermented teas.

The change of aroma in fermentation is also an important basis for judging the proper quality of black tea. The changes of aroma characteristics are as follows: grass gas, fresh fragrance, flower and fruit aroma, and sour taste. Indian scholars have systematically studied the quality detection and classification of black tea based on electronic nose technology. Nabarun Bhattacharyya *et al*. (2007) have applied mos electronic nose (MOS-EN) in the aroma monitoring in the fermentation process of broken black tea. The results showed a correlation between the results of electronic nose detection and sensory evaluation^22^. Dutta *et al*. (2003. 2011) use MOS-EN to distinguish five kinds of broken black tea with different drying degree. The results showed that the radial basis function neural network discriminant model established obtained a good recognition effect^23,24^. In summary, it is feasible to detect fermentation quality (aroma characteristics) by electronic nose technology, and use the traditional metal oxide electronic nose, which is susceptible to on-site more complex environmental factors (high temperature, high humidity) interference. Stability than the machine vision detection technology is poor.

In summary, machine vision technology can be used to detect broken black tea’s fermentation quality. But its practicability for Chinese Congou is still unknown. Compared with the CTC broken black tea processed with rolling and cutting procedures, Congou has unbroken leaf shape with more complicated and uneven color distribution. More importantly, the current studies are still focusing on the clustering discrimination and determination of the fermentation quality. The correlation, interaction, and quantitative analytical relation between image color and the quality indices (e.g. component content of pigments and sensory scores) are still undefined; besides, the prediction model is also unknown.

In order to remedy the above deficiencies, Congou black tea is chosen as the study object and the change rules of its key pigment substances are studied based on the visual sensory presentations. Linear (PLS) and nonlinear (SVM and RF) are applied in this research to establish a model for the quantitative evaluation of pigment substances (TFs, TRs, and TBs) and sensory quality. This model can realize the rapid characterization of the key quality indices during fermentation, providing a new thought and a novel technical approach for the prediction of fermentation quality and the study of special instruments.

## Conclusion

Based on machine vision technology and nonlinear modeling algorithm, this paper established a nondestructive and rapid quantitative testing method for the tea pigments and sensory quality indices during the black tea fermentation. Through spatial conversion of image colors, the study extracted 9 color variables (R, G, B, H, S, V, L, a^*^ and b^*^) as the characteristic parameters to evaluate the fermentation quality, analyzed the change rules, differences and relations of the image colors and quality indices, and established quantitative evaluation models respectively through linear and nonlinear methods. The results shows that color features and quality indices have significant differences at different periods of fermentation, but they are also significantly correlated. Compared with PLS linear model, nonlinear models (RF and SVM) can better represent the quantitative analytical relations between image information and quality indices (according to the pigment and sensory scores). The above technology can be applied to black tea automatic fermentation systems to predict and monitor quality parameters, as well as to tea processing with remarkable color changes such as withering and drying of black tea and standing of green tea, etc.

## Materials and Methods

### Experimental material

The fresh tea leaves are from Fuding variety; the tenderness of the material is a bud and a leaf. The fermentation temperature is set to 30 °C; the ambient humidity is 90%; the fermentation cycle is 300 min; 20 samples will be taken every 30 minutes; these 20 samples will be taken from different sections of the fermentation stack; totally, 220 samples will be collected; each sample weighs 100 g.

### Machine vision system and image acquisition

**Figure 1:**
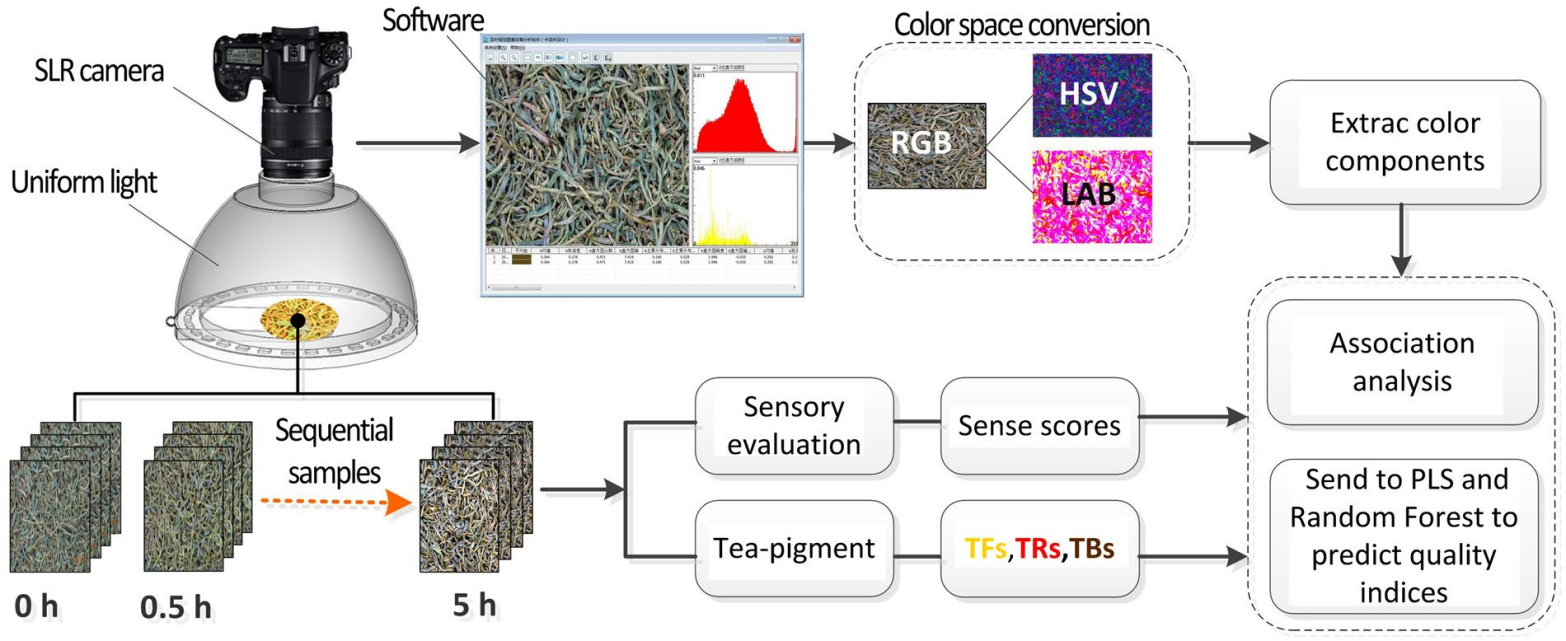
Flowchart of the algorithm employed for color measurement of tea samples.

In this research, a novel machine vision system is created, which consists of image sensor, sample pool, uniform light, and GUI software processing system. Image acquisition and data analysis are realized through the technological path of Fig. 1. Digital singles lens reflex^25^ (Canon DS60D, Japan, 18MP) is selected as image sensor; the acquisition parameters of camera^26^ are listed in Table 1. The uniform light (Sphere100, Hangzhou Flight Technology Co., Ltd, China) is chosen as the light source with an intensity of 100 lx. The required voltage is 24 V and the power is 11.3 W. The distance from the samples is 180 mm. The light emitting area is a circular area with a diameter of 106 mm. The GUI software processing system (copyright no.: 2013SR122183) is written with MATLAB 2014b (The Mathworks, Natick, MA, USA); it can be used to automatically extract the color and texture features of the image.

**Table 1:** Camera characteristics.

Characteristic	Parameter
Image size	3456 × 2304 pixels
Zoom	No zoom
Flash mode	No flash
Sensitivity	ISO-100
White balance	Fluorescence
Operation mode	Manual
Aperture av.	f/4.0
Exposure time av	1/30 s
Image type	RGB, JPEG
Macro	On
Focal length	24 mm
Resolution	72dpi

### Color feature extraction

Take 20 ± 0.5 g tea out of each sample and evenly spread it in the sample pool (Φ70 mm); embed the sample pool under the uniform light for image acquisition. A section at the size of 2000 × 1000 pixels will be automatically partitioned from the image by the software system taking pixel point (1728, 1152) as the central point; the color features of this section will then be extracted^27^.

Before analysis, the image analysis module automatically removes the shadow of the background of the image according to the threshold setting, and the shadow is formed by the underexposure of the other lower leaves caused by the light projecting onto the surface of the above table, as shown in Fig. S1(A). Digital images can be divided into grayscale images, RGB images, index images and binary images. The grayscale images are stored in a two-dimensional data matrix. The values of the elements on the matrix are the pixel grayscale, the range of which is [0, 255]. 0 means pure black and 255 means pure white. Histogram 0–30 coordinate corresponding regional distribution, as shown in Fig. S1(B) below. In this experiment, the threshold value is set to 30. That is, the pixels with gray value less than 30 are removed as shown in Fig. S1(C), then the average of the color features of remaining pixels in the ROI is extracted. (see Supplementary Fig. S1).

The following 9 color indexes are extracted through the color model conversion among RGB, HSV and CIE Lab^26^: the mean value of red channel (R), green channel (G), blue channel (B), hue (H), saturation (S), luminance (V), a component (a^*^), b component (b^*^), and lightness component (L^*^)^28^. Color transformation is based on the following formula.12

### Measurement of quality indices

The content of TFs and TRs are measured in accordance with *The Measurement of Tea Leaf’s tea pigment—High Performance Liquid Chromatography (GB/T 30483-2013)*. The samples are freeze-dried and ground, high performance liquid chromatograph (PDGU-20A3, Shimadzu Corporation, Japan) is used for the measurement. Finally, the sensory quality of each tea sample is evaluated using code review method based on the official review method for tea leaves in China (GB/T 23776-2009).

### Data processing and analysis

The linear Partial Least Squares Method (PLS), non-linear Support Vector Machine Method (SVM), and Random Forest Regression Method (RF) are used for quantitative modeling^29^. Zscore method is applied to the preprocessing of original data. Extracted from the original data with Principal Component Analysis (PCA), the feature variables are then utilized as the independent input variables of the model. Parameters applied in some literatures are used the evaluation index in this research, including Rc, Rp, RMSEC, RMSEP, Bias, RPD, SEP, and CV. In general, smaller RMSEP, SEP, CV and Bias indicate higher Rp and RPD value^30^, reflecting a more accurate and generalized model^28^. All the data are processed with Matlab 2014b 64 bit (Math Works, Natick, USA).

## Results and Discussion

### Change of color features

**Figure 2:**
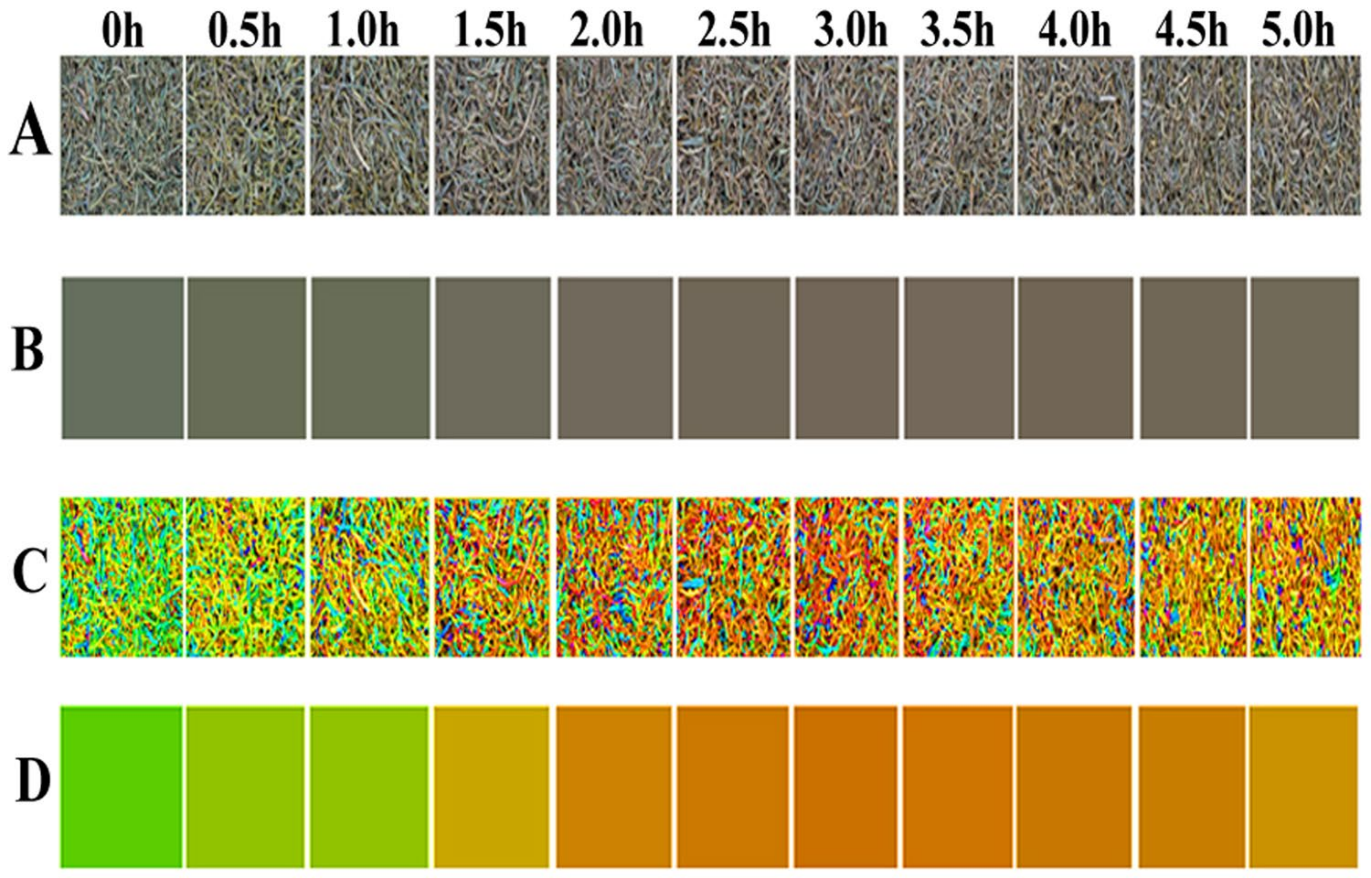
(**A**) Fermentation time images; (**B**) Average color; (**C**) Strengthened images; (**D**) Average color of the strengthened images.

In order to better study the change rules of foliage color during the fermentation, this paper first analyzed the overall visual changes. Images with different fermentation time are randomly taken and arranged according to the fermentation time order. Then the average color of the images is extracted. The results are shown in Fig. 2(A) and (B). There are certain differences of the colors with different fermentation time, which can be detected with human eyes. To better distinguish the differences, the saturation and lightness of images are strengthened. After that, the study converted RGB images to HSV color models, tripled the S channel values, doubled the V channel values, and converted the HSV to RGB images again. The results are shown in Fig. 2(C) and (D). As shown, with the process of fermentation, the foliage color is gradually changed from green to reddish yellow and then to tan. During the period of 2.5–3 h, the red has the largest presentation degree.

**Figure 3:**
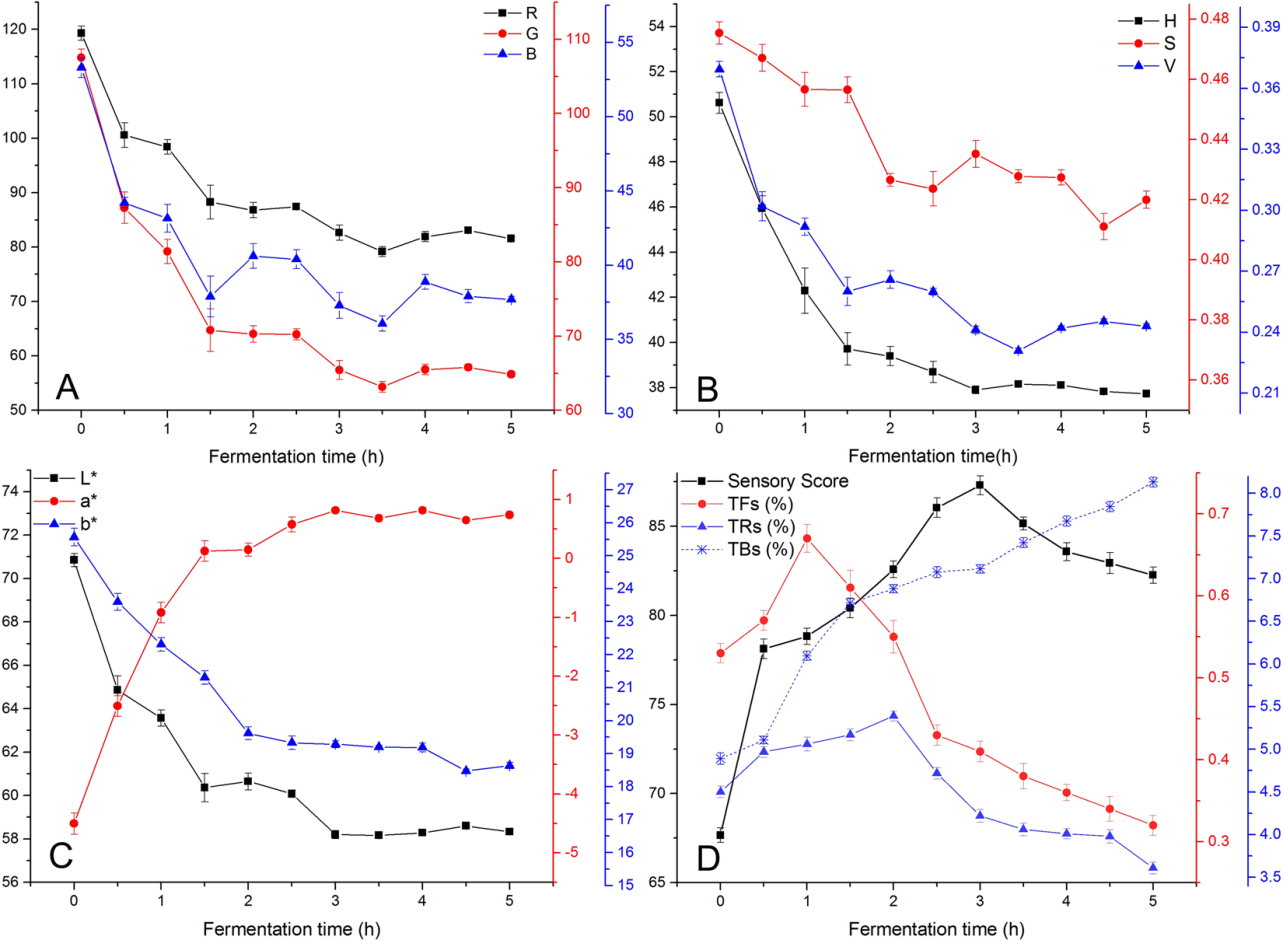
Change rules of RGB eigenvalue (**A**), HSV eigenvalue (**B**), Lab eigenvalue (**C**) and quality indices (**D**) in the fermentation.

Subsequently, parameters of different color features of the images are extracted, which presented the change rules of foliage colors from a micro perspective. The results are shown in Fig. 3. During the fermentation, all the color eigenvalues presented an overall downtrend of “fast-slow-stable” except a^*^ which is shown a general upward trend (first quick back slow). During the period of 0–1.5 h, all the color features changed dramatically, which is known as “red stain” in tea manufacture. At the time of 3 h, a^*^ and H reached the plus and minus peaks respectively, and the foliage color showed the highest degree of redness. After 3 h, the redness (R, H and a^*^) of the fermented leaves changed slightly, the b^*^ that represents the yellow blue degree is still decreasing, the “brown stain” of the fermented leaves became stronger, and the sensory quality of the black tea is going down.

### Change of quality indices

As shown in Fig. 3(D), all the quality indices observed the change rule of “rise-fall”, and the sensory quality reached the highest score at 3 h. TFs increased rapidly with the fermentation and reached the peak at the time of 1 h. Then it dropped tremendously and slowed down after the time of 2.5 h. TRs increased gradually with the fermentation and reached the peak at the time of 2 h. After that, it dropped suddenly. TBs increased continually during the whole fermentation.

### Difference analysis on quality indices and color features

A one-way ANOVA (Analysis of Variance) is conducted on the quality indices (TFs, TRs, TBs and Sensory Score) and image feature values in each phase of the fermentation. The results are shown as Table 2. The inter-group differences between the quality indices are much higher than intra-group differences, which indicated that the differences are mainly caused by different fermentation time; the significance levels are all smaller than 0.001 (Sig <0.001), which showed that the sensory quality and pigment compositions at different fermentation time are quite different.

**Table 2:** One-way ANOVA on each parameter.

Variable	Mean Square		F	Sig.
	Between Groups	Within Groups		
R	2832.183	12.77	221.791	
G	3563.196	11.106	320.83	
B	474.516	6.673	71.111	
H	339.354	0.479	708.159	
S	0.012	0.001	59.465	
V	0.031	0.001	204.957	
a*	59.186	0.066	891.014	<0.001
b*	116.925	0.291	402.03	
L*	317.844	1.424	223.24	
TFs	0.289	0.001	256.267	
TRs	6.68	0.005	1380.379	
TBs	21.896	0.008	2617.585	
Sensory Score	573.721	0.255	2252.711	

Meanwhile, the inter-group differences of color features are also greater than intra-group differences with remarkable ANOVA significance level. It showed that the extracted features at different fermentation phases are remarkably different, and the 9 color characteristic parameters are significant in recognizing different fermentation quality.

### Correlation between color features and quality indices

The correlation between quality indices (sensory scores and pigment compositions) and color feature variables of the testing samples is analyzed as shown in Table 3. The results showed that all the quality indices are remarkably correlated with color features (p < 0.01), especially with a^*^, b^*^ and L^*^ parameters under CIE Lab color model (bold in Table 3). The reason is that Lab color model has a broad color gamut. The color of “a” channel is from green to red and “b” channel is from blue to yellow, which can present colors that other color models cannot present. Especially that RGB model has too much transitional colors between blue and green, while yellow and other colors are not seen from green to red. Besides, the main features of the foliage color during the black tea fermentation are: yellowish green, reddish yellow and tan. Hence, the CIE Lab color model can present the change of the foliage color faithfully and accurately.

**Table 3:** Correlative analysis of color features and quality indices.

Variable	R	G	B	H	S	V	a*	b*	L*
G	0.995*	1							
B	0.948*	0.946*	1						
H	0.930*	0.958*	0.841*	1					
S	0.601*	0.603*	0.323*	0.698*	1				
V	0.997*	0.997*	0.965*	0.934*	0.558*	1			
a*	−0.932*	−0.959*	−0.839*	−0.999*	−0.707*	−0.935*	1		
b*	0.870*	0.876*	0.676*	0.918*	0.906*	0.846*	−0.925*	1	
L*	0.998*	0.997*	0.953*	0.941*	0.587*	0.998*	−0.942*	0.865*	1
TFs	0.546*	0.513*	0.409*	0.512*	0.566*	0.512*	−0.501*	0.618*	0.543*
TRs	0.383*	0.348*	0.287*	0.340*	0.403*	0.354*	−0.327*	0.426*	0.385*
TBs	−0.871*	−0.880*	−0.737*	−0.920*	−0.758*	−0.859*	0.921*	−0.920*	−0.879*
Sensory Score	−0.889*	−0.908*	−0.805*	−0.904*	−0.649*	−0.890*	0.915*	−0.858*	−0.888*
*TFs* ^*a*^	0.204*	0.329*	−0.238*	−0.504*	*0.062*	−0.266*	0.541*	0.260*	0.238*
*TRs* ^*a*^	−0.326*	−0.442*	−0.268*	−0.635*	*−0.082*	−0.365*	0.673*	−0.369*	−0.344*

*Correlation is significant at the 0.01 level; ^*a*^Partial correlative analysis.

TBs are significantly and positively correlated with a^*^ and significantly and negatively correlated with other color features. The correlation coefficients are obviously larger than that of TFs and TRs, which indicated that the foliage color change mainly depends on the TBs content. The higher the TBs, the darker the foliage color will be. However, according to the change of a^*^, TFs and TRs are extremely significantly and negatively correlated with a^*^, which goes against the tea chemistry theory of “the higher the TFs and the TRs, the redder the foliage will be”. The above analysis showed that, there existed a third factor that makes the correlation coefficient between a^*^ and TFs and TRs cannot truly reflect the linear degree between the two variables (that is the high sensitivity of TBs on foliage color), and conceals the influences of TFs and TRs on foliage color to some extent.

Hence, partial correlative analysis is required under the condition of controlling TBs to investigate the actual influence of the pigments on image factors^31^. The results are shown in the italics in Table 3. In the partial analysis, both TFs and TRs are significantly and positively correlated with (the correlation coefficients are 0.541 and 0.673 respectively), and negatively correlated with S; L^*^ is positively correlated with TFs and negatively correlated with TRs. Besides, sensory evaluation is extremely significantly and positively correlated with a^*^, with the correlation coefficient as much as 0.915.

According to the comprehensive analysis, foliage color has a high correlation with the content of TRs and TFs, and the color change is in accordance with the change of biochemical components. The higher the TRs, the redder the foliage will be. But too high TRs will make the foliage darker. The higher the TFs, the brighter the foliage will be, which will embody a sensory term of “red and bright”. In this study, at 3 h of the fermentation, the composition of TFs, TRs and TBs has reached the best status, when the foliage color is the redder visually and the sensory quality is the best at this time, which is in accordance with the sensory evaluation standard of black tea. Hence, color features can preferably present the dynamic change characteristics of tea pigments (TFs, TRs and TBs) during the fermentation of black tea.

### Data pretreatment and sample set division

This paper is designed to establish a correlation model between image features and quality indices during the black tea fermentation to realize a rapid nondestructive evaluation on the processing quality of black tea. During the model establishment, as the extracted image feature variables of the tea samples are diversified high-dimensional array, the paper adopted Zscore algorithm to conduct standardized conversion on the data to eliminate the influences of the dimension and order of magnitude on the model performances.

In addition, there are also certain correlations between the color feature variables between the samples (e.g. the correlation coefficient absolute values of a^*^ with other variables are all above 0.707, and its correlation with H even reached 0.999), which led to overlapped information of the variables. When involved in the modeling, this kind of redundant information may easily cause excessive risk fittings and lead to excellent calibration model but with poor predictive performances. Therefore, before establishing the predictive model, a cutting-dimension analysis of the main components should be conducted for the 9 feature variables to get 9 new uncorrelated variables (that is the number of main component factors). Then the scores of the 9 sets of independent main components are taken as the input variables of the model. This way can remove the interference information uncorrelated with the quality indices, eliminate the collinearity between the color features, and shorten the machine learning and training time.

With the quality indices scores (TFs, TRs, TBs and Sensory scores) of 220 tea samples as the model reference value, 150 samples are effectively selected as the calibration set and the rest 70 samples are selected as the prediction set through the method of Kennard-Stone (KS) based on Mahalanobis distance^32^. The actual distribution is shown as Table 4. The calibration set range of the quality indices is larger than prediction set range, which can assure the robustness of the predictive model.

**Table 4:** Descriptive statistics of quality indicator for calibration and prediction set.

Parameters	CV	Calibration set				Prediction set			
		N	Range	Mean	sd^a^	N	Range	Mean	sd^a^
TFs	0.251	150	0.279~0.723	0.466	0.117	70	0.289~0.719	0.496	0.122
TRs	0.121	150	3.570~5.500	4.527	0.542	70	3.580~5.520	4.665	0.579
TBs	0.144	150	4.939~8.292	6.999	0.992	70	4.949~8.282	6.819	1.026
Sensory score	0.063	150	67.10~88.120	81.604	5.098	70	67.230~88.110	80.821	5.233

^a^Standard deviation.

### Establishment of RF nonlinear model for each quality indices

Random forest (RF) algorithm is a highly-efficient ensemble learning method, which improves model’s prediction accuracy through the aggregation of a large number of decision trees^33^. RF contains multiple decision tree classifiers, and its output results are determined by the modes of the output results of specific decision trees. By adopting boot-strap resampling technology, RF continuously generates training samples and testing samples. Then the training samples would generate a random forest, and the prediction values of the dependent variables can be concluded by averaging the results of these trees. The ransom forest has a rapid calculation speed. It can facilitate the calculation of the nonlinear functions of variables and represent the interactions between the variables^34^. Moreover, the random forest is also not sensitive to outliers^35^. In recent years, RF algorithm has been widely applied to various industries^36^. But no literatures regarding tea leaves and image monitoring prediction have been reported.

PCs (number of principle components) and N (number of decision trees) have direct impact on the accuracy of RF model. Hence, further optimizations on N and PCs are required (within a certain range). 20 N (50–1000, with step size of 50) and 9 PCs (1–9, with step size of 1) are selected respectively to optimize parameters based on RMSEC of each quality indices model.

**Figure 4:**
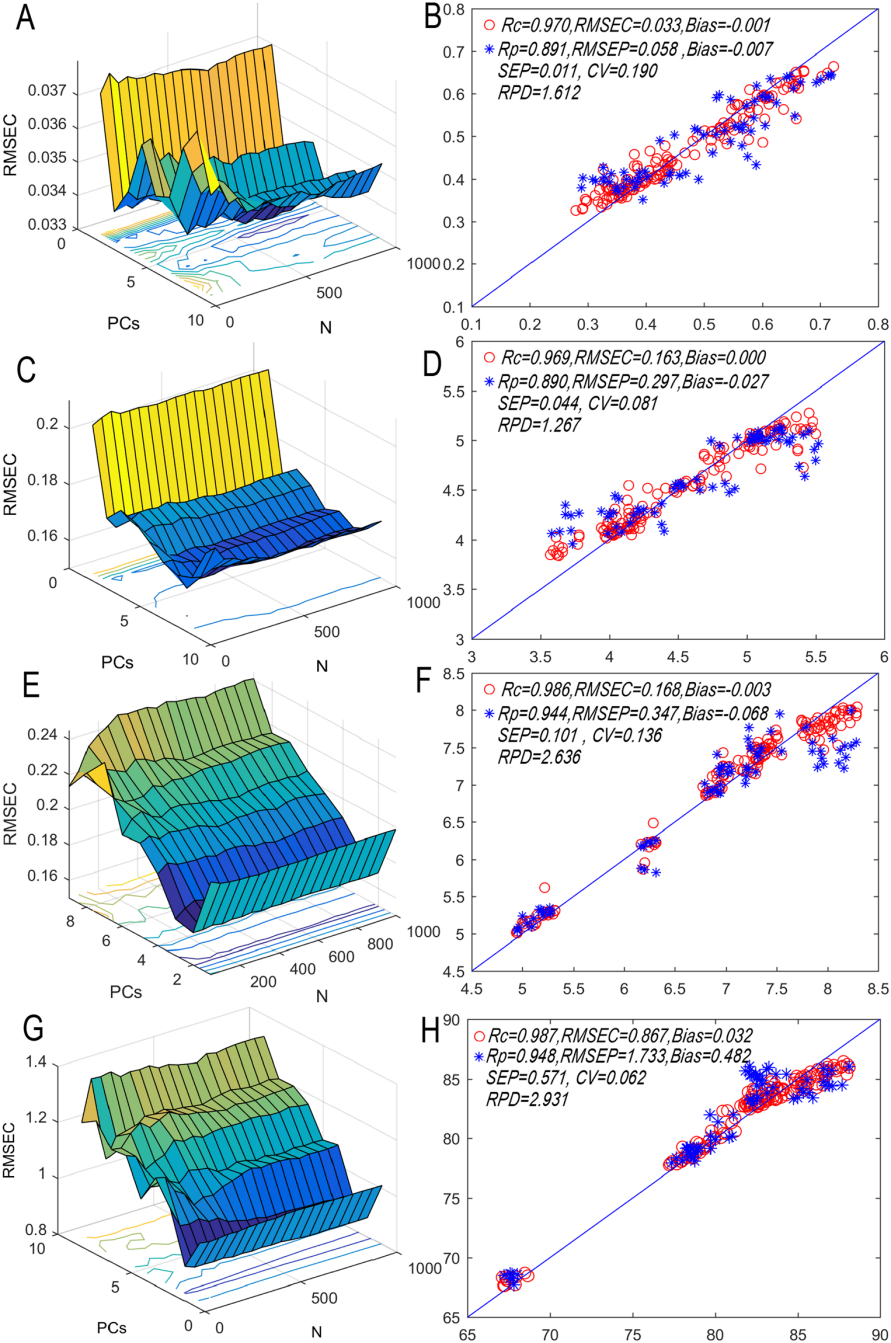
RMSEC values of each quality indice for RF models from different PCs and N((**A**) represent TFs, (**C**) represent TRs, (**E**) represent TBs and (**G**) represent sensory score), reference values versus predicted values of RF models((**B**) represent TFs, (**D**) represent TRs, (**F**) represent TBs and (**H**) represent sensory score).

The optimization results are shown as Fig. 4. In the TFs prediction model, when PCs = 6 and N = 700, RMSEC of the model reaches the minimum (0.033), Rp, RMSEP, Bias, SEP, CV and RPD of the prediction set are 0.891, 0.058, −0.007, 0.011, 0.190 and 1.612 respectively, and the relationship between the prediction value and measured value are shown as Fig. 4(B).

As shown in Fig. 4(C), in the TRs prediction model, when PCs = 7 and N = 100, the RMSEC of the model reaches the minimum (0.163), the Rp, RMSEP, Bias, SEP, CV and RPD of the prediction set are 0.890, 0.297, −0.027, 0.044, 0.081 and 1.267 respectively, and the relationship between the prediction value and measured value are shown as Fig. 4(D).

As shown in Fig. 4(E), in the TBs prediction model, when PCs = 2 and N = 200, the RMSEC of the model reaches the minimum (0.168), the Rp, RMSEP, Bias, SEP, CV and RPD of the prediction set are 0.944, 0.347, −0.068, 0.101, 0.136 and 2.636 respectively, and the relationship between the prediction value and measured value are shown as Fig. 4(F).

According to Fig. 4(G), in the sensory score prediction model, when PCs = 2 and N = 700, the RMSEC of the model reaches the minimum (0.867), the Rp, RMSEP, Bias, SEP, CV and RPD of the prediction set are 0.948, 1.733, 0.482, 0.571, 0.062 and 2.931 respectively, and the relationship between the prediction value and measured value are shown as Fig. 4(H).

### Model comparison and discussion

In order to find more optimized modeling method to realize the prediction and monitoring of quality indices, another two typical modeling methods (PLS linear model and SVM nonlinear model) are adopted respectively for performance comparison^35^. As shown in Table 5, when predicting 4 quality indices, the RMSEP of nonlinear model is obviously smaller that of PLS model, while both R and RPD are obviously higher than that of PLS model. This showed that nonlinear has better performances than linear model. The two nonlinear models have similar performances. RF modeling method is slightly superior than SVM method. The R values of the RF models of both TFs and TRs are 0.89, and the RPD values are smaller than 2 and larger than 1.0 respectively. This indicates a common performance which can distinguish level and evaluation of physical and chemical indices. The RPD values of the RF models of TBs and Sensory score are larger than 2.5, which showed the model has good prediction effect and can be used for quantitative analysis.

**Table 5:** Performances comparison between 3 models of each quality indices.

Parameters	Methods	PCs/c	N/g	Calibration set			Prediction set					
				Rc	RMSEC	Bias	Rp	RMSEP	Bias	SEP	CV	RPD
TFs	PLS	6	—	0.811	0.068	−0.001	0.795	0.075	−0.013	0.013	0.187	1.206
	SVM	0.04^**a**^	0.32^**b**^	0.881	0.055	0.001	0.886	0.056	−0.004	0.012	0.220	1.526
	RF	5	700	0.970	0.033	−0.001	0.891	0.058	−0.007	0.011	0.190	1.612
TRs	PLS	7	—	0.733	0.368	−0.006	0.752	0.388	−0.041	0.053	0.079	0.936
	SVM	5.38^**a**^	0.066^**b**^	0.853	0.282	−0.008	0.838	0.326	−0.015	0.046	0.085	1.212
	RF	7	100	0.969	0.163	0.000	0.890	0.297	−0.027	0.044	0.081	1.267
TBs	PLS	5	—	0.936	0.347	0.001	0.921	0.398	−0.010	0.101	0.137	2.346
	SVM	5.91^**a**^	0.065^**b**^	0.964	0.268	−0.049	0.940	0.294	−0.049	0.101	0.136	2.511
	RF	2	200	0.986	0.168	0.003	0.944	0.347	−0.068	0.101	0.136	2.636
Sensory score	PLS	5	—	0.929	1.887	−0.006	0.936	1.843	−0.179	0.535	0.059	2.564
	SVM	26.37^**a**^	3.39^**b**^	0.959	1.428	−0.094	0.941	1.670	0.347	0.600	0.065	2.897
	RF	2	700	0.987	0.867	0.032	0.948	1.733	0.482	0.571	0.062	2.931

^a^Represents penalty parameters (c) of SVM model; ^b^is the kernel function parameters c of SVM model.

SD, standard deviation; PCs, used latent variables; RMSEC, root mean square error of calibration; RMSEP: root mean square error of prediction; SEP, standard error of prediction; RPD, residual predictive deviation value of prediction.

Tea fermentation is usually accompanied by complicated metabolic reaction, and the changes of physical and chemical components are featured with time sequence and variability^6,9,37^. Meanwhile, as sensory evaluation is realized by distinguishing the color information of tea samples with human eyes, which then entered complicated human brain system and are integrated and comprehensively evaluated at last. Hence, the final sensory scores and colors have subjective nonlinear factors. PLS linear regression tool can hardly provide a complete solution, while nonlinear method is featured with self-learning and adjustment functions^38^, which can effectively solve complicated problems and promote the prediction capability of the model^39,40^.

## Electronic supplementary material

Supplementary Information
