# Supplementary material for: Prediction of Congou Black Tea Fermentation Quality Indices from Color Features Using Non-Linear Regression Methods
Source: Sci Rep. 2018 Jul 12;8:10535. doi: 10.1038/s41598-018-28767-2 (PMC6043511; doi:10.1038/s41598-018-28767-2)
Supplement: Supplementary file 1 — Supplementary Information [file 41598_2018_28767_MOESM1_ESM.docx]

**Supplementary Information**

**Prediction of Congou Black Tea Fermentation Quality Indices from Color Features Using Non-Linear Regression Methods**

Chunwang Dong^a*^, Gaozhen Liang^a,b^, Bin Hu ^b^, Haibo Yuan^a^, Yongwen Jiang^a^, and Hongkai Zhu ^a,c**^, Jiangtao Qi^b^

*a. Tea Research Institute Chinese Academy of Agricultural Sciences, Key Laboratory of Tea Biology and Resources Utilization, Ministry of Agriculture, Hangzhou 310008, China.*

*b. College of Mechanical and Electrical Engineering, Shihezi University, Shihezi 832003, China.*

*c. Department of Food Science, University of Copenhagen, Frederiksberg 999017, Denmark*

*Corresponding author: E-mail addresses:dongchunwang@163.com

** Corresponding author: E-mail addresses: hongkai.zhu@hotmail.com

Tel: +86-571-86653155. Fax: +86-571-86650103.

**Supplementary Figure Legends**:

**Fig.S1** Removing shadow area of sample images.

(A) Light cast shadow, (B) Shadow area in the grayscale histogram location, (C) Removing Shadow area in the grayscale histogram location


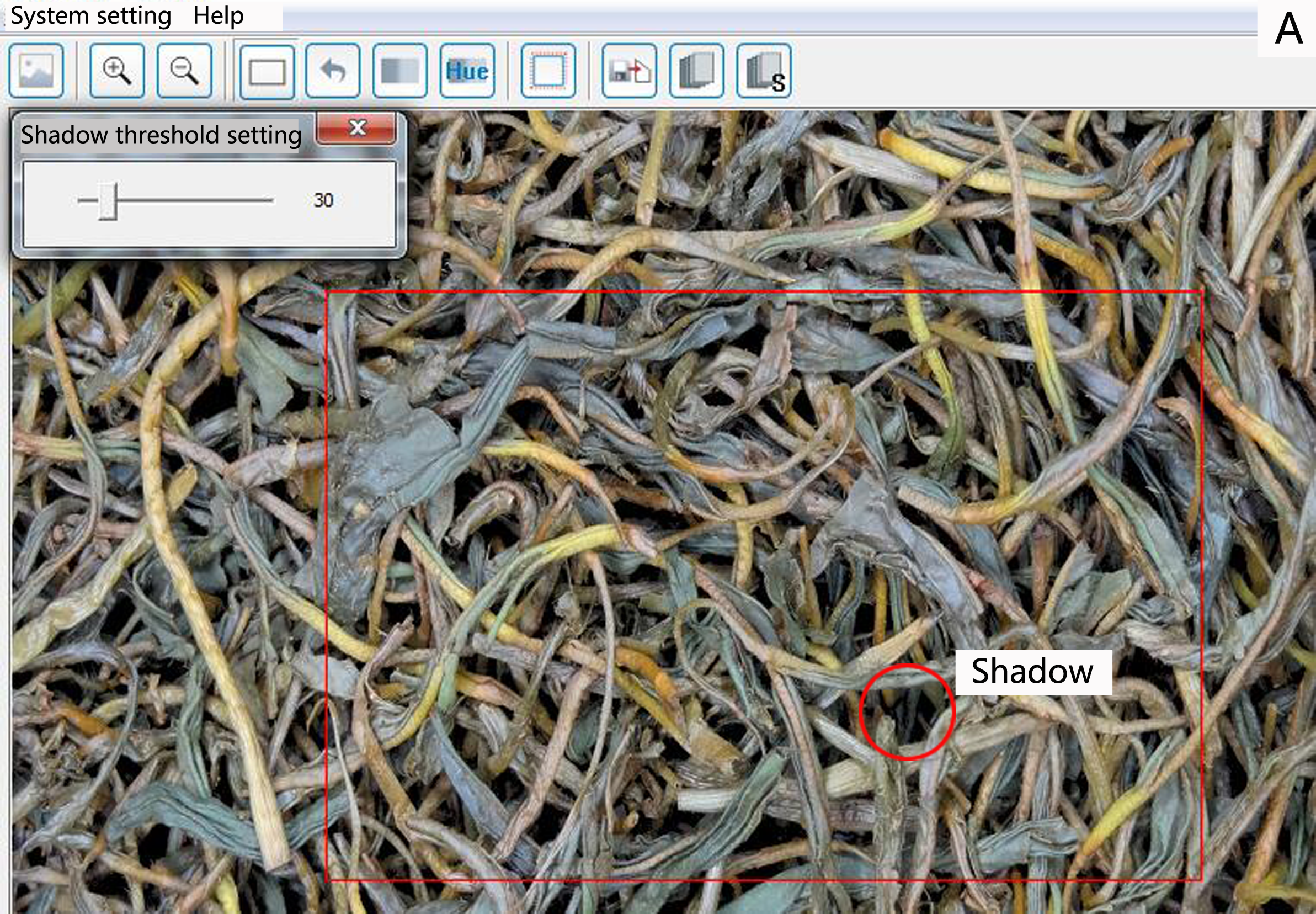


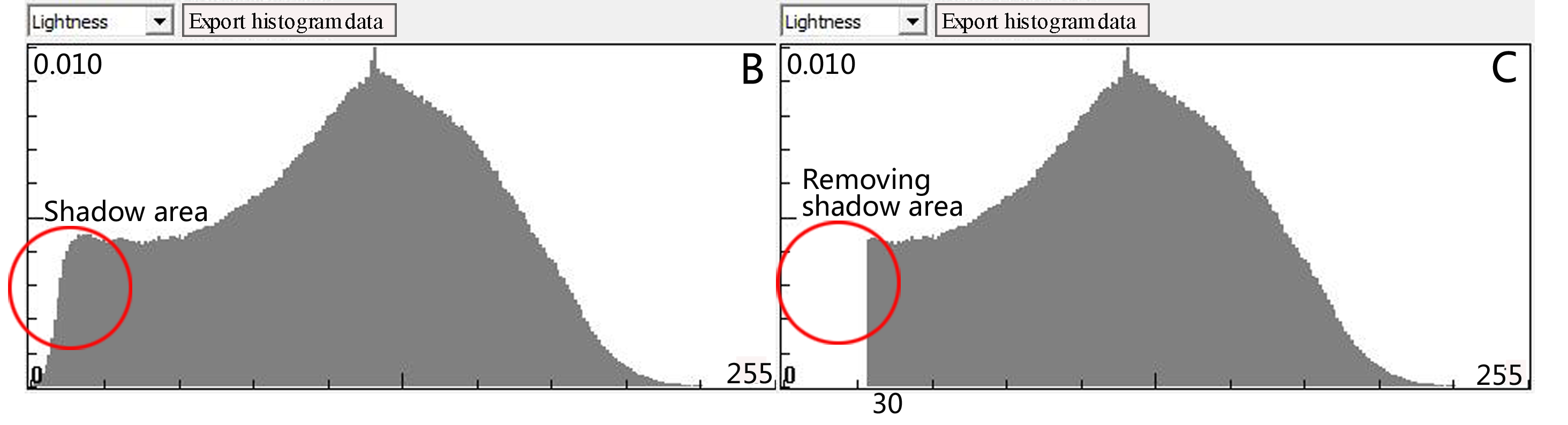


Supplementary Figure S1
